# Low-Damage Trimming of Micro Hemispherical Resonators by Chemical Etching

**DOI:** 10.3390/mi15091094

**Published:** 2024-08-29

**Authors:** Zhongzhe Zhou, Kun Lu, Yan Shi, Xiang Xi, Jiangkun Sun, Dingbang Xiao, Xuezhong Wu

**Affiliations:** College of Intelligence Science and Engineering, National University of Defense Technology, Changsha 410073, China; zhouzhongzhe2@nudt.edu.cn (Z.Z.); yanshi@nudt.edu.cn (Y.S.); xixiang@nudt.edu.cn (X.X.); sunjiangkun15@nudt.edu.cn (J.S.); dingbangxiao@nudt.edu.cn (D.X.); xzwu@nudt.edu.cn (X.W.)

**Keywords:** micro hemispherical, low-damage trimming, chemical etching

## Abstract

To enhance the performance of micro-hemispherical gyroscopes, achieving low-damage and high-surface-quality trimming is essential. This enables greater stability and reliability for the gyroscopes. Current methods for reducing frequency split often come with drawbacks such as high cost, adverse effects on the Q-factor, or surface damage. In this paper, a chemical etching trimming method is proposed to reduce frequency split in micro-hemispherical resonators. This method allows for trimming with minimal damage, while also being cost-effective and easy to implement. The theoretical basis of this method was analyzed, followed by a simulation to determine the optimal trimming range and location on the resonator. The simulated Q-factor analysis before and after trimming preliminarily validated the method’s low-damage characteristics. Ultimately, the frequency split of the resonator was reduced to below 1 Hz. Additionally, test results of the Q-factor and surface quality before and after trimming further confirmed that chemical etching offers effective low-damage trimming capabilities. The proposed method holds significant potential for improving the performance of micro-hemispherical resonator gyroscopes.

## 1. Introduction

The micro-hemispherical resonator gyroscope (MHRG) is a type of microelectromechanical gyroscope with substantial development potential and significant research value [1]. It features a compact size and low power consumption, and is also manufactured at a cost significantly lower than traditional hemispherical resonant gyroscopes [2,3]. The performance of the MHRG depends on its micro-hemispherical resonators operating in wineglass modes. Due to errors and defects in the manufacturing process, the resonators will have a frequency split $\Delta f=\left|f_{1}-f_{2}\right|$ between the theoretically equal two spindle oscillation frequencies. The wineglass mode frequency split can cause gyroscope bias drift and increased mechanical thermal noise, severely affecting the bias stability of the gyroscope [4].

In order to reduce frequency split, electrostatic and mechanical trimming has been proven useful for MHRG [5,6,7]. Frequency split can be reduced by trimming the local stiffness of the resonator through electrostatic trimming. This method, with its advantages of precise control, non-contact operation, and high flexibility, is commonly employed in microelectromechanical systems (MEMS) gyroscopes. Mechanical trimming involves the removal of material mass, permanently trimming the structural mass. This method enables frequency trimming over a wide bandwidth range without relying on high-voltage closed-loop control. Due to their small size, the micro-hemispherical resonators are typically trimmed by mass removal.

Hamelin et al. [8] proposed a eutectic trimming process for polysilicon MHRG. This method allows for a wide range of frequency split trimming. However, it has been observed that the welding trimming process may result in an uneven distribution of thermoelastic damping within the resonator, significantly impacting its quality factor. Viswanath et al. [9] accomplished mass removal from the edge of the micro-hemispherical resonator shell using micro-ultrasonic machining. Although this method achieves high precision, it has a slow removal rate and low trimming efficiency. Wang et al. [10,11] proposed a grinding release trimming process. While this method causes minimal damage to the resonator, the precision of mechanical fixtures and control is limited, resulting in lower trimming accuracy. Ion beam [12,13] and focused ion beam (FIB) trimming [14,15] are also extensively applied in this field. These methods result in low damage and high surface quality of the resonant structures post-trimming. However, they are characterized by long trimming times and expensive equipment.

Currently, laser ablation is the primary method for mechanical trimming. Bernstein et al. [16] employed high-energy density femtosecond laser ablation to investigate the frequency trimming mechanism of polycrystalline diamond micro-hemispherical resonant gyroscopes. This method achieves a wide range and high precision of frequency trimming but requires sophisticated equipment. Li et al. [17] proposed a stiffness-mass decoupling femtosecond laser trimming process, achieving frequency adjustments within a 0.1 Hz range, significantly enhancing the trimming precision of micro-hemispherical resonator. However, laser trimming commonly introduces the risk of new subsurface damage to the resonator. Current mechanical trimming methods have several issues, such as damaging the resonator, low precision, high costs, and slow trimming speeds. However, chemical etching trimming methods can address these issues effectively, and it has been explored for hemisphere and cylindrical resonators. But due to the unique structure and small size of micro-hemispherical resonators, existing trimming methods and models are not applicable. They bear many problems, such as the selection of chemical etching liquids, determination of etching positions, and control of etching ranges. Based on the existing processes, this study investigates the application of chemical etching trimming on micro-hemispherical resonators. This approach aims to achieve low-damage and high-surface-quality trimming of micro-hemispherical resonators.

Chemical etching primarily employs HF solution to etch the resonator structure, thereby achieving mass removal. The overall reaction equation for this etching process can be represented as follows:

(1) $$6HF+{SiO}_{2}=H_{2}{SiF}_{6}+H_{2}O$$

The chemical etching process virtually eliminates damage to the resonator and does not produce byproducts that could affect the surface quality or performance of the resonator. It allows the trimming process to be completed with almost no damage. The reaction rate depends on the HF concentration, a 40% HF solution achieves an etching rate of 10 nm/s, allowing rapid mass removal. Additionally, the preparation cost of HF etchant is much lower compared to equipment costs for methods such as ion beam, FIB, and lasers. These advantages give the chemical etching method significant research potential.

Consequently, a chemical etching trimming method targeted at micro-hemispherical resonators is proposed in this paper. In Section 2, the structure and operating mechanisms of the micro-hemispherical gyroscope are analyzed. The theoretical basis for trimming micro-hemispherical resonators is analyzed in Section 3. Section 4 is dedicated to presenting the simulation results and patterns of micro-hemispherical resonators chemical trimming, which are based on simulations conducted using COMSOL. Finally, Section 5 demonstrates the process and outcomes associated with micro-hemispherical resonators chemical trimming, showcasing the effectiveness and potential benefits of this method.

## 2. Structure and Operating Mechanisms

**Figure 1:**
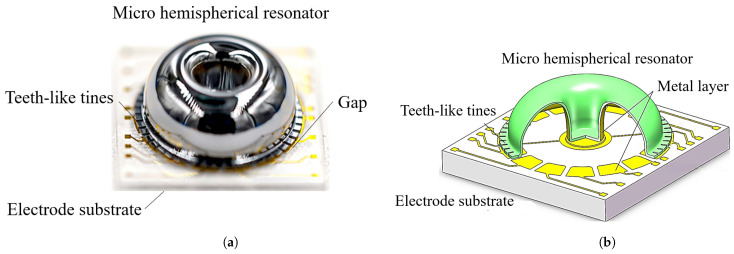
The structure of a micro-hemispherical resonator gyroscope. (**a**) Photograph of the micro-hemispherical resonator; (**b**) Model diagram of the micro-hemispherical resonator.

**Figure 2:**
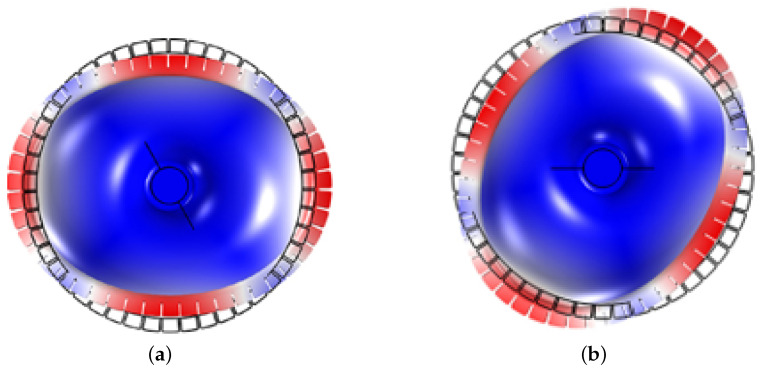
The resonators wineglass mode. (**a**) The wineglass mode 1; (**b**) The wineglass mode 2.

The micro-hemispherical resonator gyroscope studied in this paper is illustrated in Figure 1. It consists of a electrode substrate and a micro-hemispherical resonator coated with a metal layer [18]. A certain gap exists between the resonator and electrode for electrostatic excitation and detection. The micro-hemispherical resonator gyroscope is a vibratory gyroscope based on the Coriolis force, typically operating in the wineglass mode [19,20]. This wineglass mode exhibits a “circular-elliptical” four-wave nodal vibration state. It is divided into the drive mode (mode 1) and the sense mode (mode 2). The vibration patterns of these modes are oriented 45 degrees to each other, as shown in Figure 2. Changes in the vibration of the resonator structure are detected through capacitive measurements, enabling calculation of input angles and angular velocities. It is observed that a small frequency split is required for the micro-hemispherical resonator operated in the wineglass mode, necessitating the reduction of this frequency split.

**Figure 3:**
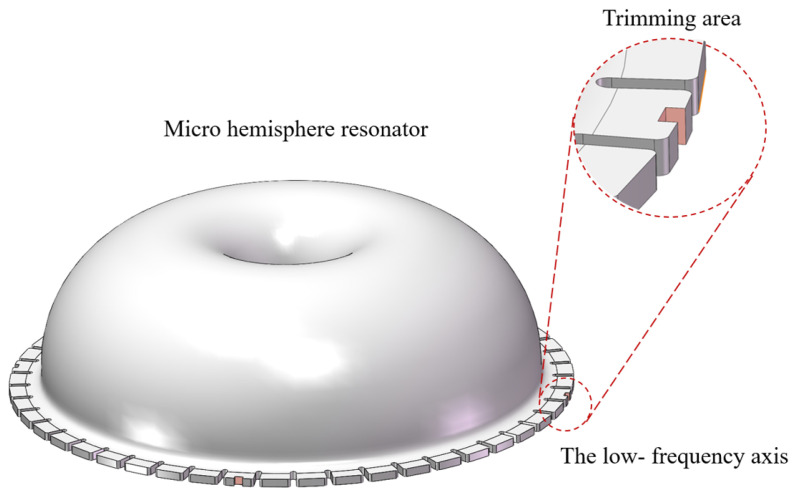
The laser ablation removal of mass on the four low-frequency axis.

Currently, the primary trimming method is laser ablation of the teeth-like tines on the low-frequency axis, as depicted in Figure 3. This method may reduce the capacitive area and introduce surface damage to the resonator potentially affecting its long-term operational stability and detection accuracy. Consequently, a chemical etching trimming method is proposed in this paper, whereby the capacitive area is preserved without the introduction of new damage.

## 3. Theoretical Analysis

The mass imbalance error of micro-hemispherical resonators can be represented in the form of a Fourier series error along the circumferential direction of the shell structure [21]:

(2) $$M\left(\varphi \right)=M_{0}+\sum_{k=1}^{\infty }M_{k}\cos k\left(\varphi -\varphi_{k}\right),$$

where $M_{0}$ represents the mass uniformly distributed along the circumferential angle; *k* denotes the harmonic number in the Fourier series, $M_{k}$ is the *k*-th harmonic of the resonator mass, and $\psi_{k}$ is the phase of $M_{k}$ relative to a defined zero direction.

**Figure 4:**
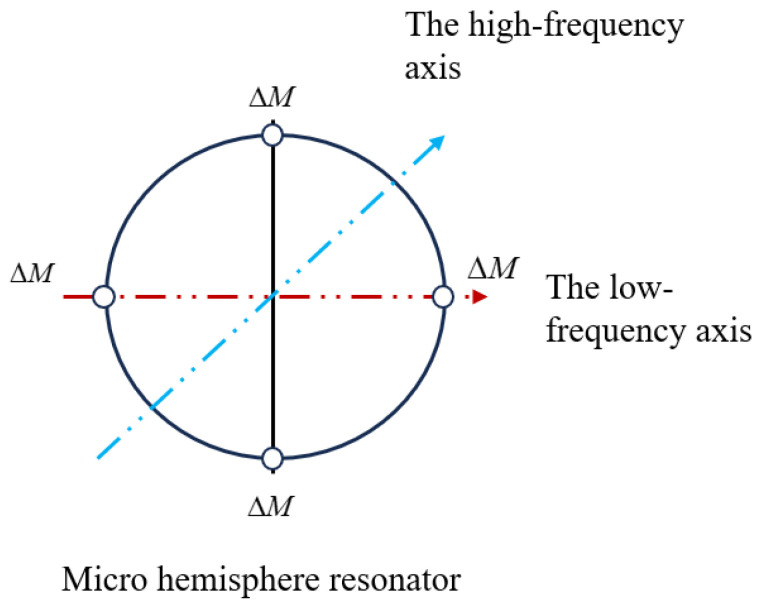
Four mass points are removed symmetrically from the edge of a micro-hemispherical resonator.

This paper primarily investigates $M_{4}$, which is the principal harmonic error leading to frequency split in micro-hemispherical resonators [22]. According to Fox [23], frequency split in micro-hemispherical resonators can be reduced by removing mass at the four low-frequency axis, as illustrated in Figure 4.

**Figure 5:**
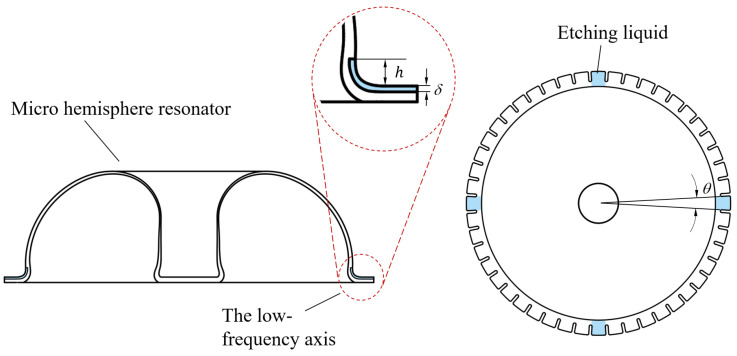
Etching liquid corrodes on four low-frequency axis; h is the height submerged into the shell, δ is the etching thickness, and θ is the angle etched on the shell.

The micro-hemisphere resonator structure, as illustrated in Figure 5, is equipped with 48 teeth-like tines around its shell [18]. Mass removal from the teeth-like tines situated on the low-frequency axis allows for frequency trimming, achievable through decoupled stiffness and mass adjustments [17]. However, due to the hydrophilic nature of quartz glass, etching liquid tends to be adsorbed onto the resonator shell. Consequently, the resonator shell is susceptible to corrosion, leading to mass-stiffness cross-coupling of wineglass modes and reducing the effectiveness of trimming.

**Figure 6:**
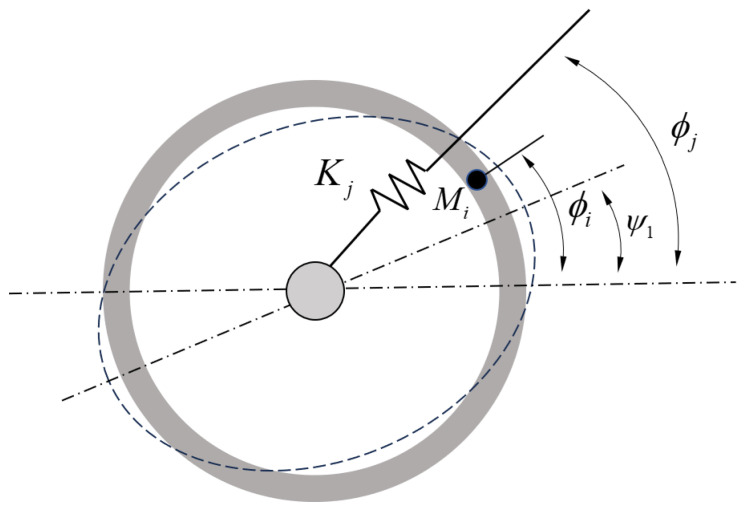
Equivalent model of non-ideal annular resonant structures.

A non-ideal ring resonator can be modeled as symmetric ring structures subjected to discrete distributions of mass points and radial spring disturbances [24], as illustrated in Figure 6. Each non-ideal mass point has a mass of $M_{i}$ and is located at $\phi_{i}$, where i=1,2,…,m. The radial stiffness distribution is represented by $N_{k}$ springs, each with a stiffness of $k_{j}$, positioned at $\phi_{j}$, where j=1,2,…,k. The natural frequencies of the non-ideal ring resonator in the wineglass mode, $\omega_{1}$ and $\omega_{2}$, and the angular positions of the mode shapes, $\psi_{1}$ and $\psi_{2}$, can be calculated using the following equations:

(3) $$\begin{array}{c} \tan4\psi_{1}==\frac{\left(1-\alpha_{2}^{2}\right)/T_{0}\sum_{i}M_{i}\sin4\phi_{k}+\alpha_{2}^{2}/4S_{0}\sum_{j}K_{j}\sin4\phi_{j}}{\left(1-\alpha_{2}^{2}\right)/T_{0}\sum_{i}M_{i}\cos4\phi_{k}+\alpha_{2}^{2}/4S_{0}\sum_{j}K_{j}\cos4\phi_{j}},\\ \psi_{2}=\psi_{1}+\frac{\pi }{4},\\ \omega_{1}^{2}=\omega_{0}^{2}\frac{1+\alpha_{2}^{2}/4S_{0}\sum_{j}K_{j}\left[1+\cos4(\phi_{j}-\psi_{1})\right]}{\left\{1+\sum_{i}\left(M_{i}/4T_{0}\right)\left[\left(1+\alpha_{2}^{2}\right)-\left(1-\alpha_{2}^{2}\right)\cos4\left(\phi_{k}-\psi_{1}\right)\right]\right\}},\\ \omega_{2}^{2}=\omega_{0}^{2}\frac{1+\alpha_{2}^{2}/4S_{0}\sum_{j}K_{j}\left[1-\cos4(\phi_{j}-\psi_{1})\right]}{\left\{1+\sum_{i}\left(M_{i}/4T_{0}\right)\left[\left(1+\alpha_{2}^{2}\right)+\left(1-\alpha_{2}^{2}\right)\cos4\left(\phi_{k}-\psi_{1}\right)\right]\right\}}, \end{array}$$

where $\alpha_{2}$ represents the ratio of radial displacement to tangential displacement amplitudes for the wineglass mode, $S_{0}$ denotes the maximum strain energy, $T_{0}$ represents the maximum kinetic energy. According to Equation (2), the frequency split vector of the non-ideal ring resonator is obtained as follows:

(4) $$\Delta_{0}e^{j4\psi_{1}}=-\lambda_{m}\sum_{i=1}^{m}M_{i}e^{j4\phi_{i}}+\lambda_{k}\sum_{i=1}^{k}K_{j}e^{j4\phi_{j}}$$

where $\Delta_{0}$ is the initial frequency split. $\lambda_{m}$ and $\lambda_{k}$ represent the mass sensitivity coefficient and stiffness sensitivity coefficient, respectively, which are associated with the dimensional parameters of the resonator. According to Li [17], when considering only mass effects, the trimming of removing a mass ${M\lambda }_{1}$ at position $\phi_{1}$ requires trimming at the following position and orientation:

(5) $$\left\{\begin{array}{c} M_{1}=-\frac{\Delta_{0}-\Delta }{\lambda_{m}},\\ \phi_{1}=\frac{4\psi_{1}+\left(2k+1\right)\pi }{4}, \end{array}\right.$$

where Δ represents the trimmed frequency split. $k=0,1,2,3,4,4\psi_{01}+\left(2k+1\right)\pi /4$ denotes the positions of four antinodal points on the low-frequency mode shape.

When considering only stiffness effects, the required trimming in stiffness magnitude and orientation are as follows:

(6) $$\left\{\begin{array}{c} K_{1}=-\frac{\Delta_{0}-\Delta }{\lambda_{k}},\\ j_{1}=\frac{4\psi_{1}+2k\pi }{4}. \end{array}\right.$$

From Equations (4) and (5), it is evident that the orientation $\phi_{1}$ for mass trimming and $j_{1}$ for stiffness trimming should differ by pi/4 angle. When changes in mass and stiffness are coupled at the same orientation, reducing local mass also decreases stiffness at that orientation, this will result in a counteracting trimming effect.

Given the difficulty in characterizing the coupling between stiffness and mass in actual trimming process, it is necessary to conduct simulations of trimming ranges. These simulations are required to determine the extent of the impact that stiffness and mass have during the trimming process.

## 4. Simulation Experiment

The frequency split of the wineglass mode in micro-hemispherical resonators was simulated using COMSOL Multiphysics 6.0 software. The geometric parameters and material properties of the micro-hemispherical resonators are detailed in Table 1. By adjusting the local density of the model, a frequency split is introduced in the micro-hemispherical resonators. Simulations are performed with fixed constraints imposed at the base of the resonator pillar. The wineglass mode exhibits two eigen frequencies: $f_{1}$ = 6416.5 Hz and $f_{2}$ = 6425.4 Hz, resulting in an initial frequency split of 8.9 Hz.

**Figure 7:**
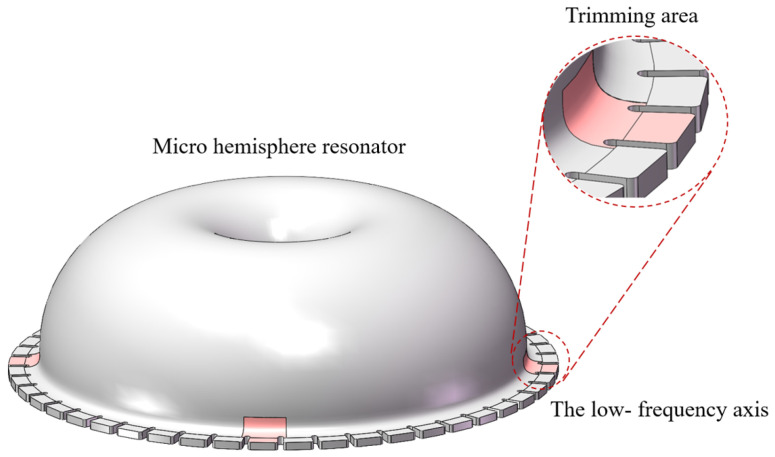
The simulation model after mass removal at the four low-frequency axis.

The target frequency split is within 1 Hz. An analysis of the changes in the resonator frequency split is conducted based on varying the parameters *h*, θ, and δ as illustrated in Figure 5, and the simulated structure is depicted in Figure 7. According to practical circumstances, $0\;\mu m\leq h\leq 600\;\mu m,\;0^{\circ }\leq \theta \leq 18^{\circ },\;0\;\mu m\leq \delta \leq 100$ μm.

**Figure 8:**
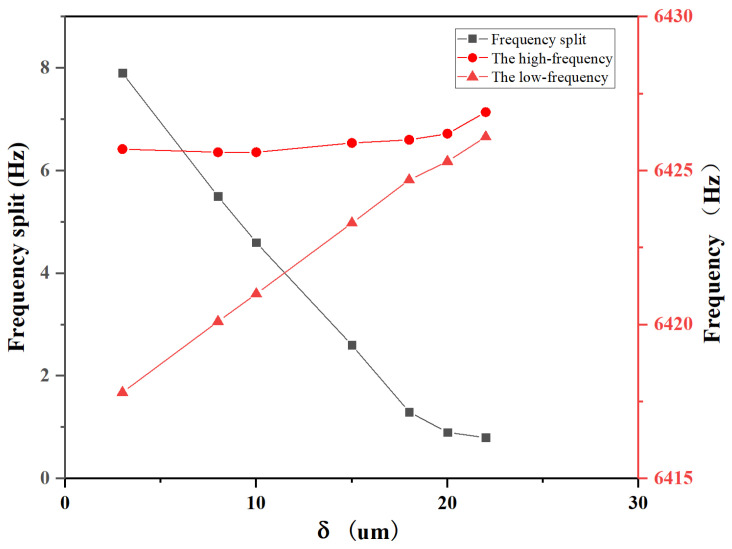
For θ=0 and h=0, the variation of frequency split and frequency with respect to δ.

The special case of θ=0 and h=0 is initially analyzed. As depicted in Figure 8, simulation results reveal that with an increasing δ, a continuous decrease in the frequency split is observed, while the low-frequency experiences a significant increase and the high-frequency undergoes a slight change. This indicates that a distinct mass removal trimming effect is achieved with minimal impact on stiffness.When the etching solution is applied only to the resonator’s teeth-like tines, the trimming process decouples stiffness and mass, and it results in mass trimming.

**Figure 9:**
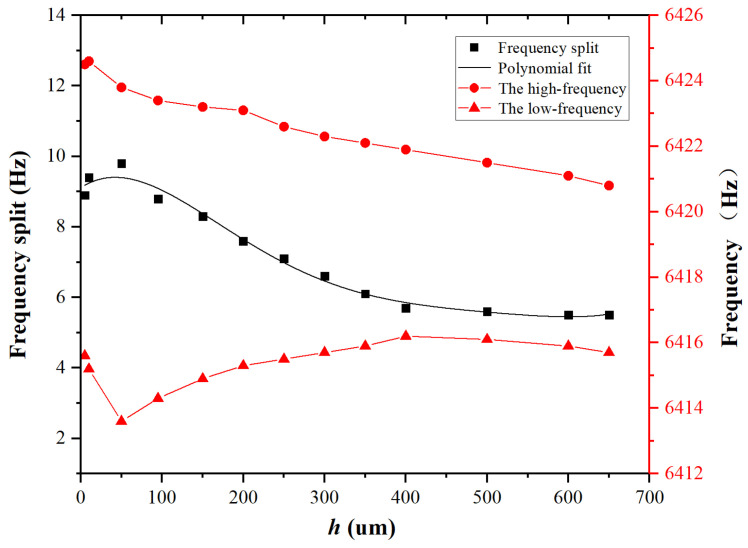
For $\theta =18^{\circ }$ and δ=3 μm, the variation of frequency split and frequency with respect to *h*.

Next, with $\theta =18^{\circ }$ and δ=3 μm set, simulations were conducted to analyze the variation of frequency split at different *h*. The simulation results are presented in Figure 9. It has been observed that an increase in *h* at the low-frequency axis position enhances the reduction of frequency split more effectively. However, once *h* reaches a certain threshold, the trimming effect gradually stabilizes. Further analysis reveals that when h≤100 μm, stiffness exerts a greater influence than mass, significantly weakening the trimming effect of mass removal and resulting in an increase in frequency split. When h>100 μm, the influence of mass removal on the resonator becomes dominant, gradually enhancing the trimming effect. When h>400 μm, the effects of stiffness and mass removal trimming are observed to stabilize, with a gradual attenuation in the enhancement of the trimming effect.

**Figure 10:**
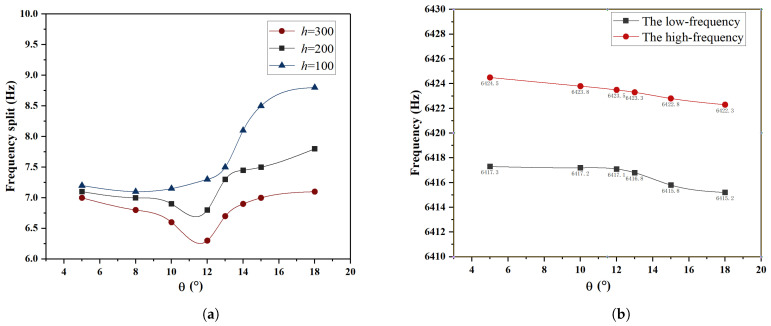
For *h* = 100 μm, 200 μm, 300 μm, and δ=3 μm, the variation of frequency split and frequency with respect to θ. (**a**) The frequency split varies with respect to θ; (**b**) the frequency varies with respect to θ.

Under the conditions of *h* = 100 μm, 200 μm, 300 μm, and δ=3 μm, the influence of different θ on frequency split was analyzed, and the results are shown in Figure 10. In Figure 10a, it can be observed that around $\theta =12^{\circ }$, an optimal trimming effect is achieved, resulting in the smallest frequency split. As depicted in Figure 10b, when $\theta <12^{\circ }$, a decrease in the high-frequency is observed, while the low-frequency remains slightly higher than the initial frequency. At this point, a weaker inhibitory effect of stiffness on the mass trimming of the low-frequency axis is exhibited, but a decrease in stiffness contributes to lowering the high-frequency, thereby increasing the efficiency of trimming. Subsequently, when $\theta >12^{\circ }$, the influence of stiffness on the mass trimming of the low-frequency axis increases, leading to a significant decrease in the low-frequency and a decrease in trimming efficiency.

**Figure 11:**
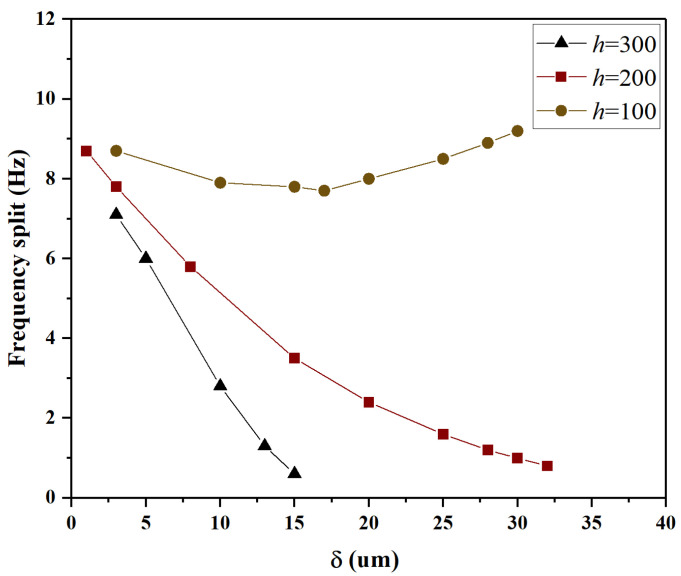
For *h* = 100 μm, 200 μm, 300 μm, and $\theta =18^{\circ }$, the variation of frequency split with respect to *h*.

Lastly, under the conditions of *h* = 100 μm, 200 μm, 300 μm, and $\theta =18^{\circ }$, the influence of different δ on frequency split was analyzed, and the results are presented in Figure 11. For *h* = 200 μm, and 300 μm, with the increase in δ, it is observed that the frequency split continuously decreases, eventually reaching below 1 Hz. However, when h=100 μm, the minimum frequency split that can be achieved is only 7.2 Hz. Analysis shows that when h<100 μm, stiffness and mass are highly coupled. In this case, the trimming effects cancel each other out, it becomes difficult to reduce the frequency split by trimming. This indicates that when *h* is below a certain threshold, the required trimming effect cannot be achieved.

Simultaneously, simulations were conducted to analyze changes in the resonator’s quality factor before and after trimming. The simulation results are shown in Table 2. Due to a slight decrease in frequency split after trimming, the quality factor also changed to some extent, but overall, it did not have a significant impact.

Based on the simulation results, it is recommended to control the range of the etching solution during chemical etching trimming when *h* = 400 μm and $\theta =12^{\circ }$. At this point, it is observed that the mass effect significantly exceeds the stiffness effect, resulting in a greater amount of mass removed compared to trimming solely on the teeth-like tines, thus achieving the highest trimming efficiency.

**Figure 12:**
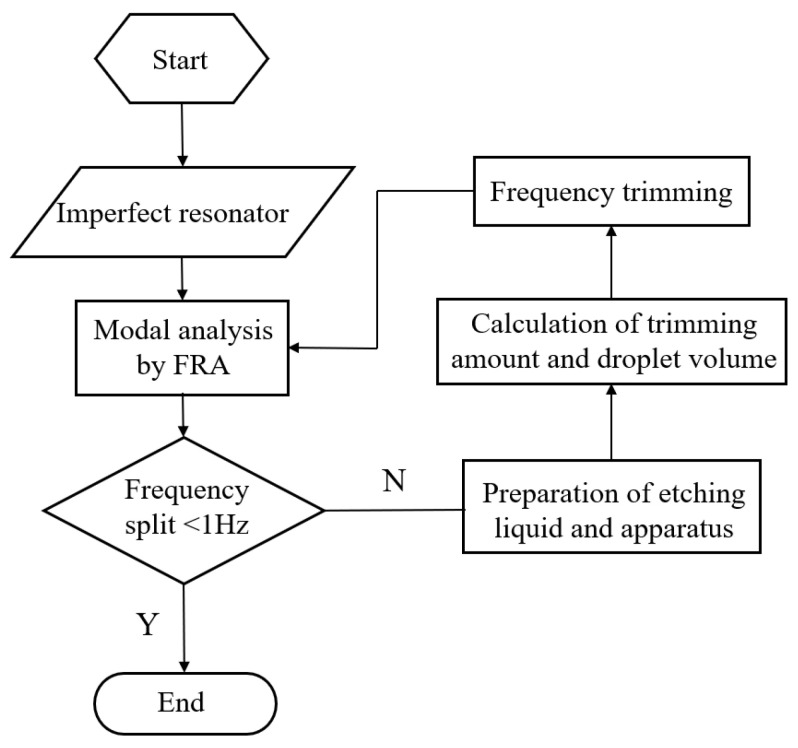
The process of chemical etching trimming.

According to theoretical analysis and simulation results, the trimming process depicted in Figure 12 was obtained. Initially, the low-frequency axis position of the resonator was tested using frequency response analyzer (FRA) and marked. Next, the etching solution and apparatus are prepared. The trimming amount and droplet volume are calculated based on the frequency split, and the etching height *h* is controlled by regulating the droplet volume. Finally, trimming is performed and tested again until the frequency split is below 1 Hz. And the etching solution used in the experiment is an HF solution. The etching rates of fused quartz vary with different concentrations of HF solution, as shown in Table 3. If the concentration of the etching solution is too low, the trimming time will be excessively long, and the diffusion range of the etching solution will be wider. Conversely, if the concentration is too high, the removal rate will be too fast, making it difficult to control the quality of removal. Therefore, this experiment primarily uses a 40% concentration of HF solution, which ensures both rapid trimming and precise control over the quality of removal.

## 5. Frequency Trimming Procedure and Results

**Figure 13:**
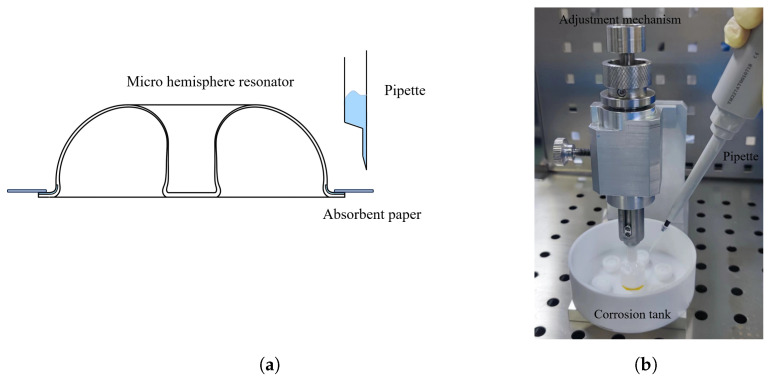
The experimental apparatus. (**a**) Schematic diagram; (**b**) The actual apparatus.

The experimental setup is depicted in Figure 13. Figure 13a features a schematic diagram of the experiment, where indirect contact is made between the micro-hemispherical resonator and the etching liquid. This contact leads to the spreading of the etching liquid over the surface of the shell, potentially corroding up to half of the shell. To mitigate this, absorbent paper is placed on the teeth of the resonator’s low-frequency axis. The etching liquid is then deposited onto the absorbent paper using a pipette. This indirect method of application restricts the spread of the etching liquid, enabling more precise adjustment of the trimming location. Figure 13b illustrates the actual apparatus, which primarily consists of a corrosion tank, an adjustment mechanism, and a pipette. Resonators with initial frequency splits of 7.19 Hz, 7.61 Hz, and 8.93 Hz were selected for the experiments, and were designated as H01, H02, and H03, respectively.

**Figure 14:**
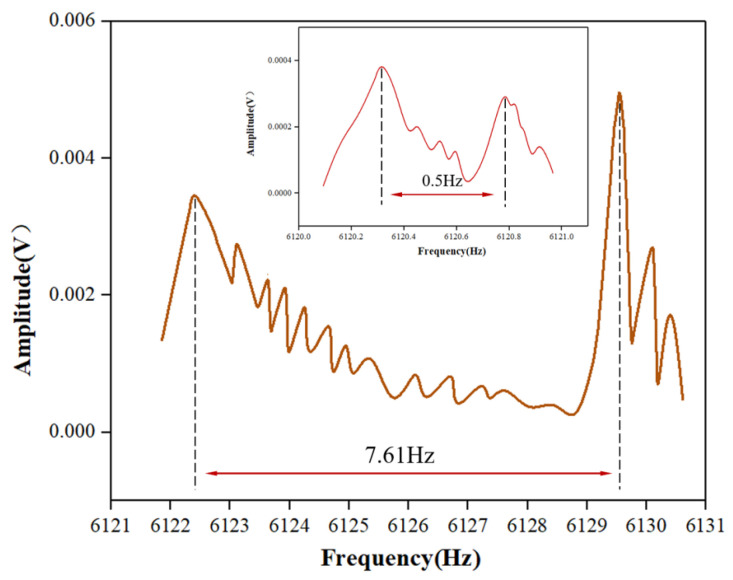
The frequency split change for H02 before and after trimming.

**Figure 15:**
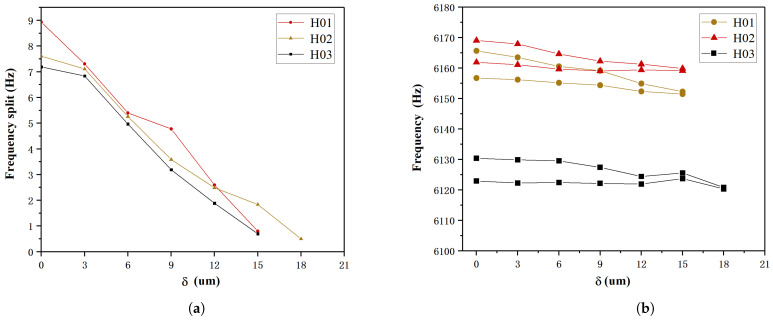
Frequency split and frequency variation during the trimming process. (**a**) Variation of frequency split with δ; (**b**) Variation of frequency with δ.

**Figure 16:**
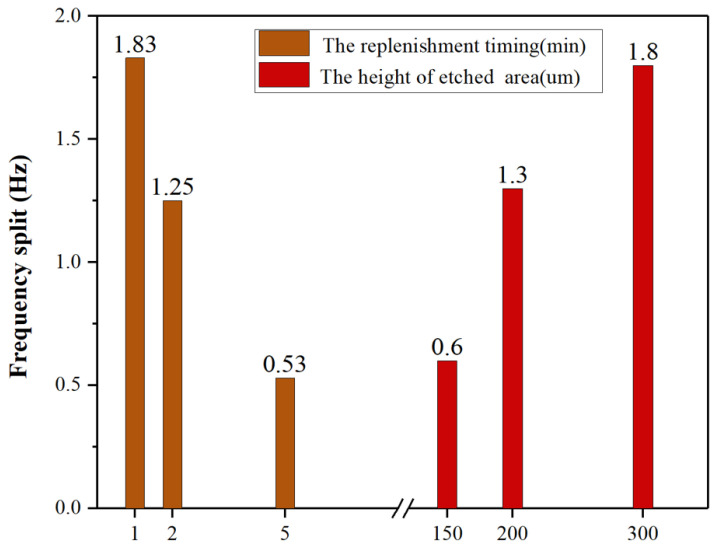
Comparison of trimming efficiency for different replenishment times and different etched area heights.

The frequency split of the resonators ultimately reached 0.7 Hz, 0.5 Hz, and 0.8 Hz. The test results for H02, with the smallest frequency split after trimming, are shown in Figure 14. Minimal change was observed in the low-frequency, as shown in Figure 15, while the high-frequency continuously decreased, consistent with simulation results for $\theta <12^{\circ }$. During the trimming process of the resonators, control over the replenishment timing allowed for some regulation of the trimming area, as shown in Figure 16, the results represent the average of various experiments, and it closely match the simulation results with *h* = 300 μm, 200 μm, 150 μm, respectively. Increasing the droplet volume appropriately can enhance the wicking height of the etching liquid on the casing, thereby improving trimming efficiency. However, excessive replenishment caused the etching liquid to spread beyond the intended area, exceeding the low-frequency axis range and adversely affecting the trimming effect.

**Figure 17:**
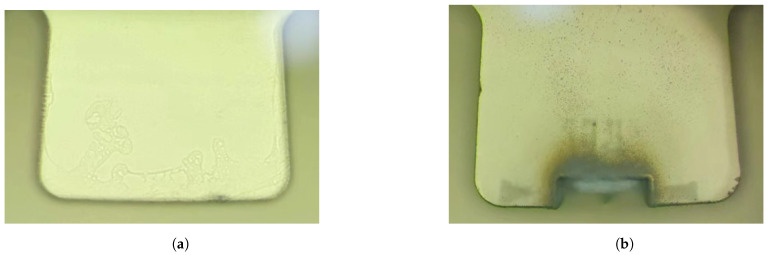
Images of resonator teeth-like tines surfaces after trimming. (**a**) Images of resonator tooth surfaces after laser ablation trimming; (**b**) Images of resonator tooth surfaces after chemical etching trimming.

Comparing the resonator teeth-like tines surfaces trimmed by laser ablation and chemical etching, as shown in Figure 17, reveals significant differences. Laser ablation trimming causes greater damage to the resonator and results in substantial dust contamination. In contrast, chemical etching trimming causes far less damage and has no dust contamination issues. The only drawback is a slight increase in surface roughness due to uneven corrosion from etching solution bubbles.

**Figure 18:**
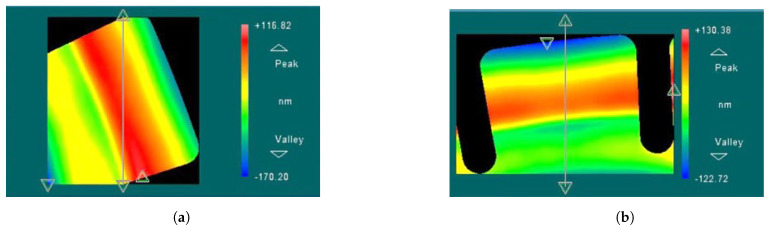
The surface roughness of the teeth-like tines before and after trimming. (**a**) Before trimming, RMS=49.832; (**b**) After trimming, RMS=54.009 nm.

The surface roughness of the teeth-like tines before and after trimming was measured using a white light interferometer, as shown in Figure 18. The results indicate that there is minimal change in the surface roughness of the teeth before and after trimming, suggesting that chemical etching trimming has a negligible impact on the surface quality of the resonator.

**Figure 19:**
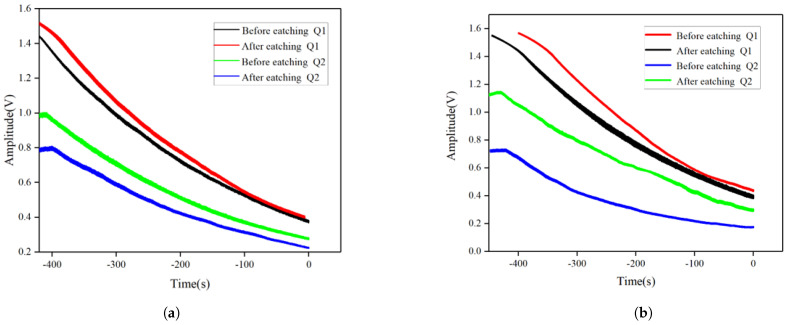
Amplitude decay curves of the resonator before and after trimming. (**a**) Amplitude Decay Curves of H01 Before and After Trimming; (**b**) Amplitude Decay Curves of H02 Before and After Trimming.

Simultaneous testing was conducted on the changes in quality factor of H01 and H02, with the results shown in Table 4 below. The resonator’s amplitude decay curve was measured using a laser vibrometer to obtain the Q factor of the resonator. The test curve is shown in Figure 19. The change in decay time before and after trimming is minimal, the Q-factor of the resonator slightly decreases due to the reduction in frequency. It was observed that the chemical etching trimming method did not significantly decrease the quality factor of the resonator.

In summary, the experiments validated the feasibility of chemical etching trimming. Frequency splits of three resonators were reduced to below 1 Hz, with the results aligning closely with simulations. Microscopic observation and Q-factor testing confirmed the low-damage nature of the chemical etching trimming process.

## 6. Conclusions

For the first time, frequency split trimming of the wineglass mode of micro-hemispherical resonators has been successfully achieved using chemical etching. It is observed that chemical etching has the potential to accomplish frequency split trimming without affecting the quality factor. Through the utilization of chemical etching, the frequency split can be reduced to less than 1 Hz. Additionally, chemical etching trimming is considered to be more cost-effective and easier to implement compared to methods such as laser ablation and grinding. However, chemical etching trimming also encounters challenges in controlling the extent of etching, which requires further exploration.

## Figures and Tables

**Table 1:** Geometric Parameters and Material Properties.

Parameters	Amount	Units
Yang’s modulus	73.1	GPa
Poisson’s ratio	0.17	-
Density	2203	kg/m^3^
Shell height	3.8	mm
Shell diameter	12	mm
Tooth length	0.5	mm
Tooth thickness	0.22	mm

**Table 2:** Simulation results of quality factor changes before and after etching.

Status	$f_{1}$ (Hz)	$Q_{1}$	$f_{2}$ (Hz)	$Q_{2}$
Before etching	6416.9	$4.21\times 10^{7}$	6425.8	$4.2\times 10^{7}$
After etching	6412.4	$4.05\times 10^{7}$	6413	$4.1\times 10^{7}$

**Table 3:** Etching rates of HF solutions at different content.

Content	Rate (nm/s)
1%	0.1
10%	1
30%	5
40%	10
50%	30

**Table 4:** The comparison of the quality factor of H02 before and after chemical etching.

Status	H01 $Q_{1}$	H01 $Q_{2}$	H02 $Q_{1}$	H02 $Q_{2}$
Before eatching	$6.21\times 10^{6}$	$6.18\times 10^{6}$	$5.93\times 10^{6}$	$5.61\times 10^{6}$
After eatching	$6.14\times 10^{6}$	$6.13\times 10^{6}$	$5.83\times 10^{6}$	$5.52\times 10^{6}$

## Data Availability

The data that support the findings of this study are available from the corresponding author, Zhongzhe Zhou, upon reasonable request.

## Abbreviations

The following abbreviations are used in this manuscript:

MHRG	Micro Hemispherical Resonator Gyroscope
FRA	Frequency Response Analyzer
FIB	Focused Ion Beam
